# Fibrosis-4 (FIB-4) Index Is Associated With Skeletal Muscle Mass in Japanese Patients With Type 2 Diabetes: A Single-Center Retrospective Cross-Sectional Study

**DOI:** 10.7759/cureus.107785

**Published:** 2026-04-27

**Authors:** Hiroyuki Ito, Chizuko Yukawa, Toshiko Mori, Chiaki I, Mizuki Matsushita, Suzuko Matsumoto, Hideyuki Inoue, Shinichi Antoku, Tomoko Yamasaki, Kenichi Nishio, Sayuri Miura

**Affiliations:** 1 Diabetes, Metabolism and Kidney Disease, Edogawa Hospital, Tokyo, JPN; 2 Laboratory, Edogawa Hospital, Tokyo, JPN

**Keywords:** fib-4, fibrosis-4 (fib-4) score, liver fibrosis, non-elderly patients, skeletal muscle mass, skeletal muscle mass index, smi, type 2 diabetes

## Abstract

Introduction

Liver fibrosis is increasingly recognized as a metabolic complication that may contribute to reduced skeletal muscle mass in type 2 diabetes. While the fibrosis-4 (FIB-4) index is a widely used non-invasive liver fibrosis marker, its clinical utility for assessing muscle mass remains unclear. Chronic inflammation and insulin resistance, which drive liver fibrosis, are also implicated in muscle degradation via the liver-muscle axis. We investigated the association between the FIB-4 index and low skeletal muscle mass index (SMI) in Japanese patients with type 2 diabetes, focusing on the influence of age.

Methods

This retrospective, cross-sectional study included 476 outpatients. SMI was assessed via bioelectrical impedance analysis; low SMI was defined by the Asian Working Group for Sarcopenia (AWGS) 2019 criteria. The relationship between the FIB-4 index and low SMI was evaluated using multiple logistic regression analyses and the Cochran-Mantel-Haenszel test, stratified by age (<65 vs. ≥65 years).

Results

Low SMI was identified in 111 (23%) of participants. In multivariable models adjusted for sex, BMI, and cerebrovascular disease, FIB-4 index was significantly associated with low SMI (OR 1.65, 95% CI 1.07-2.56, P=0.02). However, when age was added as a covariate, this association lost statistical significance (OR 0.83, 95% CI 0.48-1.42, P=0.50). Subgroup analysis revealed that in patients aged <65 years, those with FIB-4 index ≥1.3 had a significantly higher prevalence of low SMI (χ^2^=4.44, P=0.04), whereas no such association was observed in those aged ≥65 years.

Conclusions

The apparent association between the FIB-4 index and low SMI is predominantly driven by the confounding effect of age. While exploratory analyses suggest a potential signal in non-elderly patients (<65 years), the independent clinical utility of the FIB-4 index for assessing muscle mass remains uncertain and requires further prospective validation.

## Introduction

Sarcopenia, characterized by decreased skeletal muscle mass and function, is a critical comorbidity in patients with type 2 diabetes [1,2] that significantly increases the risk of falls, fractures, and mortality [3-9]. Clarifying the underlying mechanisms and establishing early screening strategies are urgent clinical priorities. The prevalence of sarcopenia in type 2 diabetes is driven by multiple factors, including insulin deficiency, chronic hyperglycemia, inflammation, oxidative stress, and neuropathy [2,10].

Concurrently, it is known that patients with type 2 diabetes have a high comorbidity rate of fatty liver disease [11]. Steatotic liver disease involves excessive fat accumulation in the liver, which may progress to steatohepatitis, fibrosis, cirrhosis, or hepatocellular carcinoma [12]. Assessing the degree of liver fibrosis is essential for understanding disease severity and determining an appropriate treatment strategy. For this reason, risk stratification using the fibrosis-4 (FIB-4) index is strongly recommended in adult patients with type 2 diabetes [11]. The FIB-4 index is easy to integrate into routine clinical practice, as it is calculated from common laboratory parameters: age, aspartate aminotransferase (AST), alanine aminotransferase (ALT), and platelet count [13].

Advanced liver fibrosis not only causes structural and functional liver abnormalities and increases cancer risk, but also affects systemic metabolism. It has been reported that advanced liver fibrosis is a major cause of protein-energy malnutrition (PEM) [14-16]. PEM is a state of chronic malnutrition, which can result in a decrease in skeletal muscle mass and function, i.e., sarcopenia. Therefore, it is plausible that in patients with type 2 diabetes, progression of liver fibrosis may accelerate the decrease in skeletal muscle mass via PEM, even at a stage that has not yet progressed to liver cirrhosis. Previous studies have reported extensively on the high prevalence of sarcopenia in patients with liver cirrhosis and the association between the severity of liver disease and sarcopenia [17,18]. Furthermore, the association between the FIB-4 index and decreased skeletal muscle mass has also been suggested in general elderly individuals with no history of liver disease treatment [19,20], indicating the possibility that liver fibrosis affects skeletal muscle mass. However, research that has thoroughly investigated the association between liver fibrosis and skeletal muscle mass at a pre-cirrhotic stage in a specific population like patients with type 2 diabetes is limited [21,22].

Although the diagnosis of sarcopenia requires the assessment of muscle strength (e.g., handgrip strength) in addition to muscle mass [23], performing these physical performance tests in busy outpatient clinics can be challenging due to time constraints and the need for specific equipment. In contrast, the FIB-4 index offers a significant clinical advantage as it can be automatically calculated from routine blood tests without additional cost or effort. Therefore, establishing a relationship between the FIB-4 index and skeletal muscle mass could position the FIB-4 index as a readily available screening tool for high-risk patients.

The primary objective of this study was to assess the independent association between the FIB-4 index and skeletal muscle mass index (SMI) in patients with type 2 diabetes. Given that age is inherently included in the FIB-4 formula, our secondary, exploratory objective was to evaluate this association across different age strata. Through these analyses, we aimed to clarify the clinical complexities of using the FIB-4 index as a marker for assessing muscle mass decline in a real-world diabetic population.

## Materials and methods

Study subjects

**Figure 1:**
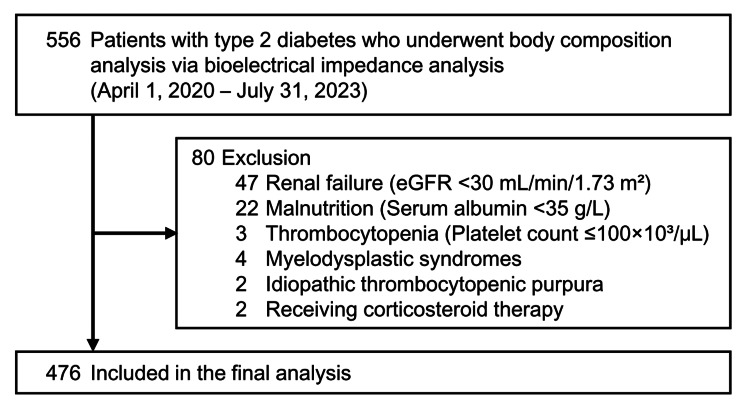
Participant flow diagram Of the 556 patients with type 2 diabetes who underwent bioelectrical impedance analysis, 80 were excluded based on the specific criteria. The final analysis included 476 patients. eGFR: estimated glomerular filtration rate.

From April 1, 2020, to July 31, 2023, we enrolled 556 consecutive adult outpatients with type 2 diabetes who attended the Department of Diabetes, Metabolism, and Kidney Disease at Edogawa Hospital, Tokyo, Japan. These patients had no evidence of liver cirrhosis and underwent body composition analysis via bioelectrical impedance analysis (BIA). To minimize potential confounding effects on skeletal muscle mass and the FIB-4 index, we excluded patients with renal failure (estimated glomerular filtration rate or eGFR <30 mL/min/1.73 m²; n=47), malnutrition (serum albumin <35 g/L; n=22), thrombocytopenia (platelet count ≤100 × 103/μL; n=3), myelodysplastic syndromes (n=4), idiopathic thrombocytopenic purpura (n=2), and those receiving corticosteroid therapy (n=2). As a result, 476 patients were included in the final analysis (Figure 1).

Methods

This was a retrospective, cross-sectional study; therefore, causal relationships could not be determined. All clinical data were extracted from the electronic medical records of the Edogawa Hospital. The reporting of this study conforms to the Strengthening the Reporting of Observational Studies in Epidemiology (STROBE) guidelines [24]. Information regarding diabetes duration, comorbidities, and medication use was obtained through a detailed review of electronic medical records (chart review). Comorbidities were defined based on physician diagnoses documented in the medical history. Medication use was defined as the current prescription at the time of the BIA measurement.

Skeletal muscle mass was assessed using a multi-frequency bioelectrical impedance analyzer (InBody S10, InBody Japan Inc., Tokyo, Japan). Measurements were performed in a fasting state, after urination and rest, at a standard room temperature of 20-25°C. All BIA measurements were performed by trained clinical staff following standardized institutional protocols to ensure consistency and reliability. The primary parameter of interest was the SMI, calculated using the appendicular skeletal muscle mass. According to the 2019 Asian Working Group for Sarcopenia (AWGS) criteria [23], low SMI was defined as <7.0 kg/m² in men and <5.7 kg/m² in women in this study.

The FIB-4 index was calculated for each patient using the following formula [13]:

$$FIB\mathrm{-}4 = \frac{Age \ (years) \times AST \ (U/L)}{Platelet \ count \ (10^3/\mu L) \times \sqrt{ALT \ (U/L)}}$$

A value of ≥1.3 was considered indicative of increased risk of liver fibrosis [25,26].

Information on lifestyle and comorbidities was collected from the medical records. A current drinker was defined as a person consuming >20 g of ethanol equivalent per day. Although the metabolic dysfunction-associated steatotic liver disease (MASLD) criteria define thresholds of 30 g/day for men and 20 g/day for women [27] we adopted this strictly uniform cutoff (>20 g/day) for both sexes based on the “Health Japan 21” guidelines by the Ministry of Health, Labour and Welfare, which define appropriate alcohol consumption as approximately 20 g/day [28]. Specifically, previous epidemiological studies in the Japanese population have consistently demonstrated that alcohol consumption of approximately 20 g/day is associated with the lowest risk of mortality and better metabolic profiles [29,30].

Hypertension was defined as a systolic blood pressure (SBP) of ≥140 mmHg and/or a diastolic blood pressure (DBP) of ≥90 mmHg. Participants currently using antihypertensive medications were also classified as being positive for hypertension. Hyper low-density lipoprotein (LDL) cholesterolemia was defined as a serum LDL-cholesterol (LDL-C) concentration of ≥3.62 mmol/L (140 mg/dL) or the current use of statins or ezetimibe. Hypo high-density lipoprotein (HDL) cholesterolemia was defined as a serum HDL-cholesterol (HDL-C) concentration of <1.03 mmol/L (40 mg/dL).

Microvascular complications of diabetes were assessed as follows: diabetic retinopathy was evaluated by an ophthalmologist. Albuminuria was defined as a urinary albumin-to-creatinine ratio of ≥30 mg/gCr in a spot urine sample. Diabetic peripheral neuropathy was diagnosed based on the diagnostic criteria proposed by the Diabetic Neuropathy Study Group in Japan [31], which require the presence of at least two of the following: subjective symptoms such as numbness in both lower extremities, reduced or absent Achilles tendon reflexes, and diminished vibration perception at the medial malleolus.

Macrovascular complications were defined as follows: cerebrovascular disease was diagnosed based on neurological findings at onset and confirmed by brain CT or MRI. Coronary heart disease was identified by a documented history of myocardial infarction or angina pectoris, or diagnosis via coronary angiography. Peripheral artery disease was defined by the presence of ischemic symptoms in the lower limbs and confirmation of arterial stenosis or occlusion by ultrasonography.

Ethical approval

This study was conducted in accordance with the principles of the Declaration of Helsinki. The study protocol was approved by the Ethics Committee of Edogawa Hospital, and written informed consent was waived because the data analyzed in this retrospective study were anonymous and based on information stored within the hospital (approval number: 2024-20, approval date: July 4, 2024). The trial was registered with the University Hospital Medical Information Network Clinical Trials Registry (UMIN-CTR; identifier UMIN000058647).

Statistical analyses

Given the retrospective nature of this study, no a priori sample size calculation was performed; instead, all consecutive eligible patients during the study period were included. Continuous variables are presented as the mean ± standard deviation or median (interquartile range [IQR]). Categorical variables are presented as percentages, and the 95% confidence intervals (CIs) for the prevalence of low SMI were calculated based on the binomial distribution. The association between SMI and the FIB-4 index was assessed using Pearson’s correlation coefficient. Linear regression analysis using a least-squares model was used to evaluate the associations between the SMI and clinical parameters. To clarify the association between the FIB-4 index and low SMI, we established two analytical approaches based on our research hypotheses. First, to test whether the association between the FIB-4 index and low SMI remained significant after controlling for the categorical effect of age, we performed the Cochran-Mantel-Haenszel (CMH) test stratified by age groups (<65 vs. ≥65 years). The age stratification threshold of 65 years was chosen in accordance with the standard definition of the elderly population in Japan. Second, to determine the association between the FIB-4 index and low SMI after adjusting for potential confounders, we conducted multiple logistic regression analyses using three different models. Covariates for these models were selected a priori based on their established clinical relevance to skeletal muscle mass and diabetes complications, as well as their significance in univariate analyses.

To address the conceptual collinearity between age and the FIB-4 index, we systematically constructed three models: Model 1 was constructed to include key clinical confounders (age, sex, and BMI) as well as other variables that showed a significant association with SMI in univariate analysis, ensuring a comprehensive adjustment. Furthermore, because age is both a strong independent risk factor for muscle mass decline and a mathematical component of the FIB-4 formula, we explicitly addressed its profound confounding effect to assess the independent effect of the FIB-4 index. Model 2 excluded age and antidiabetic medications closely associated with age (sulfonylureas, thiazolidinediones, dipeptidyl peptidase-4 (DPP-4) inhibitors; sodium-glucose cotransporter 2 (SGLT2) inhibitors; glucagon-like peptide-1 (GLP-1) receptor agonists) to avoid multicollinearity. Covariates were selected based on their established clinical relevance to skeletal muscle mass and diabetes complications to isolate the non-age-related effect of the FIB-4 index. Model 3 focused on clinically relevant factors potentially associated with SMI, including sex, duration of diabetes, BMI, presence of cerebrovascular disease, hemoglobin level, serum albumin level, and the FIB-4 index.

Model 1 was considered the primary multivariable model to adjust for potential confounders including age. In the multiple logistic regression analyses, the FIB-4 index was entered as a continuous variable. The categorical cutoff (≥1.3) was used for the stratified analysis (CMH test) and descriptive statistics. For the main regression models, patients with missing data for any of the included variables were excluded (complete-case analysis), and no data imputation methods were applied. Among the covariates in the primary model, the duration of diabetes had the highest missingness, involving 33 (6.9%) participants. While excluding cases with missing data may slightly reduce statistical power, it was considered unlikely to introduce substantial bias given the relatively low proportion of missing data (<10%) across key variables. Consequently, the analytic sample size varied across models due to missing values in specific covariates (n=413 for Models 1 and 2; n=435 for Model 3).

To evaluate the potential non-linear relationship between the FIB-4 index and low SMI, restricted cubic spline (RCS) analyses were performed. This method was employed to flexibly model and visualize the continuous dose-response relationship without assuming a strict linear association. The RCS model was specified with three knots placed at the 10th, 50th, and 90th percentiles of the FIB-4 distribution. The models were adjusted for sex to account for the baseline differences in muscle mass between genders. These analyses were conducted to visualize the relationship between the FIB-4 index and the predicted probability of low SMI within each age subgroup (<65 years and ≥65 years). The Wald test was used to assess the significance of the overall association and non-linearity. The presence of influential observations was evaluated using Cook’s distance. Multicollinearity, particularly between age and the FIB-4 index, was assessed using the variance inflation factor (VIF). While a VIF value of <10 was considered to indicate the absence of significant statistical multicollinearity, we acknowledge that conceptual collinearity inherently exists since age is a mathematical component of the FIB-4 formula.

Statistical significance was set at p <0.05 (two-tailed). JMP version 12.2.0 (SAS Institute, Cary, NC, USA) and EZR version 1.68 (Saitama Medical Center, Jichi Medical University, Saitama, Japan), which is a graphical user interface for R (The R Foundation for Statistical Computing, Vienna, Austria), were used to perform all analyses.

## Results

Clinical characteristics of the study population

The clinical characteristics of the study population are summarized in Table 1.

**Table 1:** Clinical characteristics of the study participants FIB-4, Fibrosis-4; DPP, dipeptidyl peptidase; SGLT, sodium-glucose cotransporter; GLP, glucagon-like peptide; LDL, low-density lipoprotein; HDL, high-density lipoprotein; eGFR, estimated glomerular filtration rate; SMI, skeletal muscle mass index; AST, aspartate aminotransferase; ALT, alanine transaminase.

Variables	N	N (%) /mean ± SD
Men (%)	476	310 (65%)
Age (years)	476	65±14
Duration of diabetes (years)	443	12±11
Current drinker (%)	473	157 (33%)
Smoking history (%)	472	241 (51%)
Body mass index (kg/m^2^)	476	25.9±4.7
Body mass index ≥25.0 kg/m^2^ (%)	476	256 (54%)
Antidiabetic medication (%)		
Sulfonylureas	476	40 (8%)
Metformin	476	226 (47%)
Thiazolidinediones	476	25 (5%)
DPP-4 inhibitors	476	182 (38%)
SGLT2 inhibitors	476	173 (36%)
GLP-1 receptor agonists	476	76 (16%)
Insulin preparations	476	126 (26%)
Hypertension (%)	476	364 (76%)
Hyper LDL-cholesterolemia (%)	476	303 (64%)
Hypo HDL-cholesterolemia (%)	476	88 (18%)
Diabetic retinopathy (%)	437	104 (24%)
Diabetic peripheral neuropathy (%)	422	173 (41%)
Albuminuria (%)	475	230 (48%)
Cerebrovascular disease (%)	476	53 (11%)
Coronary heart disease (%)	476	82 (17%)
Peripheral artery disease (%)	476	21 (4%)
Hemoglobin (g/L)	476	142±17
Serum albumin (g/L)	467	43±3
LDL-cholesterol (mmol/L)	465	2.71±0.36
HDL-cholesterol (mmol/L)	465	1.34±0.36
Uric acid (μmol/L)	464	297.4±77.3
eGFR (mL/min/1.73 m^2^)	476	75±23
HbA1c (%)	475	8.6±2.2
SMI (kg/m^2^)	476	7.2±1.3
Low SMI (%)	476	111 (23%)
AST (U/L)	476	25±14
ALT (U/L)	476	28±27
Platelet count (10³/μL)	476	242±69
FIB-4 index	476	1.47±0.76
FIB-4 ≥1.3 (%)	476	254 (53%)

The mean age was 65 years, with a range of 22 to 92 years. The first quartile, median, and third quartile were 54, 68, and 76 years, respectively. Among men, the age ranged from 22 to 92 years, with a mean of 62 years, and the first quartile, median, and third quartile were 53, 64, and 73 years, respectively. Among women, the age ranged from 23 to 92 years, with a mean of 68 years, and the first quartile, median, and third quartile were 60, 71, and 78 years, respectively.

Association between SMI and the FIB-4 index

**Figure 2:**
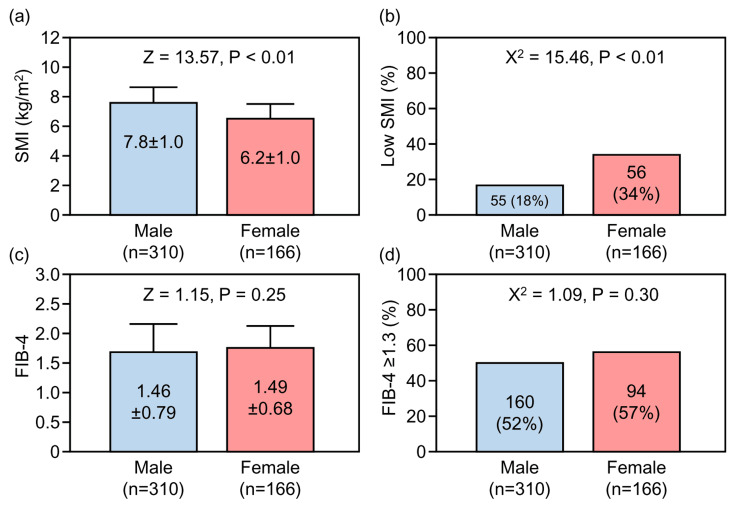
Comparison of skeletal muscle mass index (SMI) and the FIB-4 index, stratified by sex (a) Mean SMI; (b) Prevalence of low SMI; (c) Mean fibrosis-4 (the FIB-4 index); (d) Prevalence of the FIB-4 index ≥1.3. Error bars in (a) and (c) represent standard deviations.

Figure 2 illustrates the mean values of SMI and the FIB-4 index, as well as the prevalence of low SMI and the FIB-4 index ≥1.3, stratified by sex.

The overall mean SMI was 7.2 kg/m² (Table 1), but a significant sex difference was observed, with women showing significantly lower values than men (Figure 2a).

The prevalence of low SMI was 111 (23%) overall (Table 1) and was significantly higher in women than in men (Figure 2b). There was no significant sex difference in the FIB-4 index values (Figure 2c), nor in the proportion of individuals with the FIB-4 index ≥1.3, which indicates an increased risk of liver fibrosis (Figure 2d).

**Figure 3:**
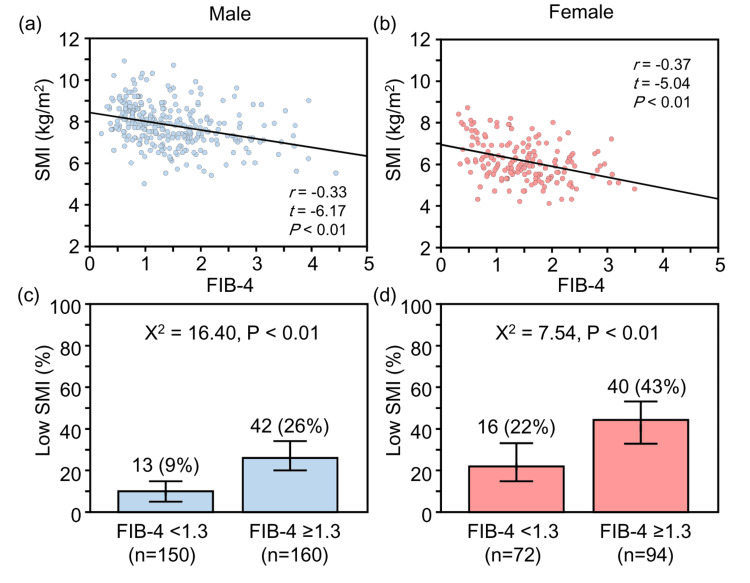
Association between SMI and the FIB-4 index (a) Correlation between skeletal muscle mass index (SMI) and fibrosis-4 (the FIB-4 index) in men; (b) Correlation between SMI and the FIB-4 index in women; (c) Prevalence of low SMI by the FIB-4 index category in men; (d) Prevalence of low SMI by the FIB-4 index category in women. Error bars represent the 95% confidence intervals based on the binomial distribution.

Figure 3 shows the association between SMI and the FIB-4 index stratified by sex.

In both men (Figure 3a) and women (Figure 3b), SMI was significantly and negatively correlated with the FIB-4 index. Furthermore, the prevalence of low SMI was significantly higher in the group with the FIB-4 index ≥1.3 than in the group with the FIB-4 index <1.3, for both men (42 (26%) (95% CI: 20-34%) vs. 13 (9%) (95% CI: 5-14%); Figure 3c) and women (40 (43%) (95% CI: 33-53%) vs. 16 (22%) (95% CI: 14-33%); Figure 3d).

Age-stratified analysis

**Figure 4:**
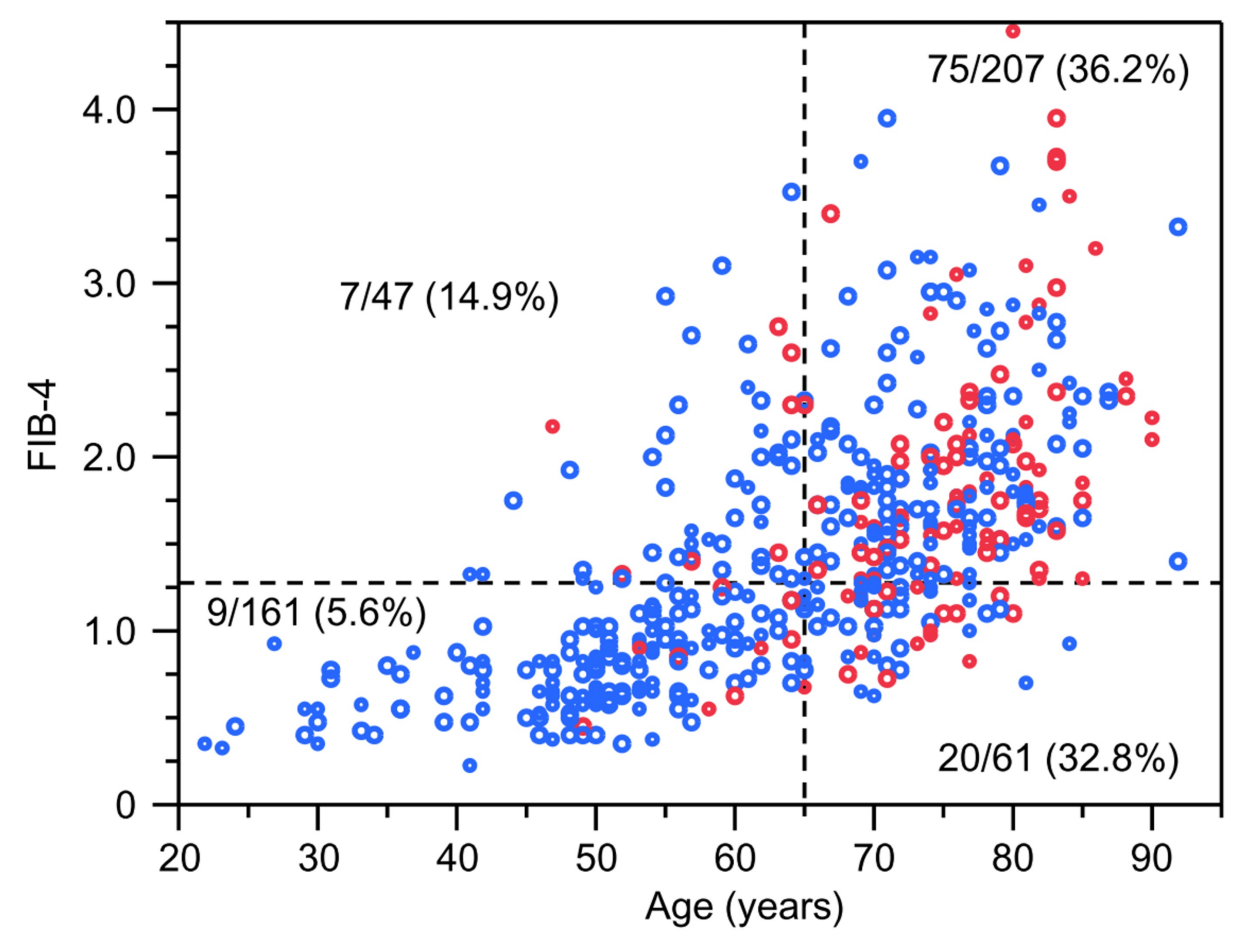
Scatter plot of age versus fibrosis-4 (FIB-4) index values in all participants (n=476) Red circles denote individuals with low skeletal muscle mass index (SMI); blue circles denote those without low SMI.

Figure 4 illustrates the relationship among low SMI, age, and the FIB-4 index.

Among individuals younger than 65 years, the prevalence of low SMI was nine (5.6%) out of 161 individuals in the FIB-4 index <1.3 group and seven (14.9%) out of 47 individuals in the FIB-4 index ≥1.3 group.

In contrast, among individuals aged 65 years and older, the prevalence of low SMI was 20 (32.8%) out of 61 individuals in the FIB-4 <1.3 group and 75 (36.2%) out of 207 individuals in the FIB-4 index ≥1.3 group.

The CMH test, adjusted for age group, yielded a common odds ratio of 1.43 (95% CI: 0.84-2.43; χ^2^=1.51, P=0.22), indicating no statistically significant association between low SMI and the FIB-4 index overall. However, in the subgroup aged <65 years, the prevalence of low SMI was significantly higher in the FIB-4 index ≥1.3 group compared to the <1.3 group (χ^2^=4.44, P=0.04), whereas no significant difference was observed between the FIB-4 index groups in those aged ≥65 years. Furthermore, a logistic regression analysis testing the interaction between the FIB-4 index category and age group yielded a P value of 0.13 (χ^2^=1.44). Although this did not reach statistical significance, it is consistent with our finding that the association between the FIB-4 index and low SMI appears to be more pronounced in the younger age group.

**Figure 5:**
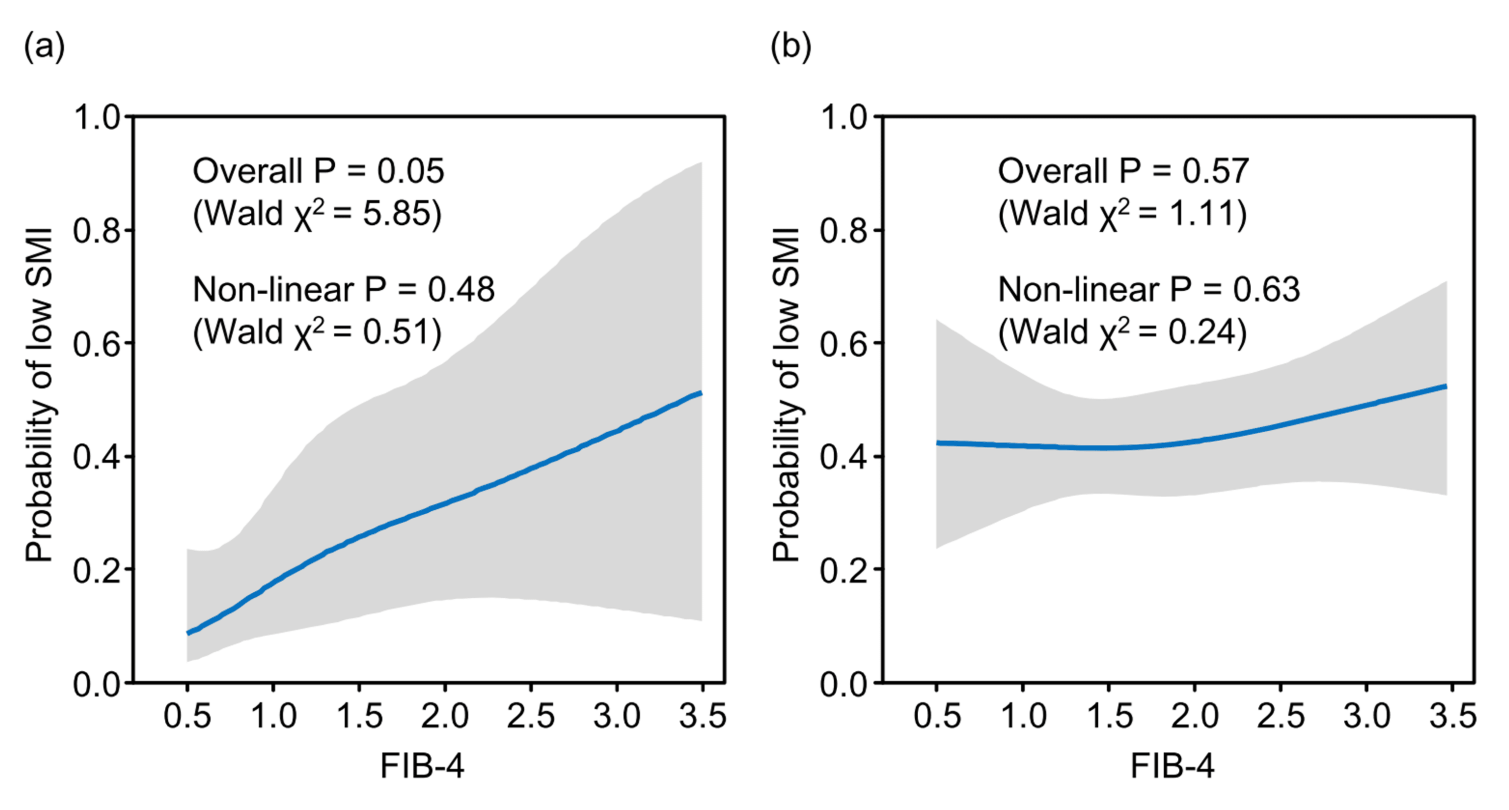
Restricted cubic spline plots of the association between the FIB-4 index and the probability of low SMI, stratified by age (a) Non-elderly group (<65 years); (b) Elderly group (≥65 years). The solid blue line represents the estimated probability, and the gray shaded area indicates the 95% confidence interval.

To visually explore this relationship, RCS analyses stratified by age group and adjusted for sex were performed. In the non-elderly group (<65 years), a marginal positive linear trend was observed, with the probability of low SMI increasing as the FIB-4 index values rose (overall association: Wald χ^2^=5.85, P=0.05; Figure 5a).

This association did not deviate significantly from linearity (non-linear component: Wald χ^2^=0.51, P=0.48). In contrast, no significant association was observed in the elderly group (≥65 years) for either the overall effect (Wald χ^2^=1.11, P=0.57; Figure 5b) or the non-linear component (Wald χ^2^=0.24, P=0.63).

Logistic regression analyses of factors associated with low SMI

Table 2 summarizes the patient background factors associated with low SMI.

**Table 2:** Association between low skeletal muscle mass index (SMI) and clinical characteristics OR, odds ratio; CI, confidence interval; NA, not analyzed; DPP, dipeptidyl peptidase; SGLT, sodium-glucose cotransporter; GLP, glucagon-like peptide; LDL, low-density lipoprotein; HDL, high-density lipoprotein; eGFR, estimated glomerular filtration rate. For categorical variables, the reference group is the absence of the condition (e.g., non-drinker, no history of disease), unless otherwise specified.

Variables	Single regression		Multiple regression					
	OR (95% CI)	P	Model 1 (n=413)		Model 2 (n=413)		Model 3 (n=435)	
			OR (95% CI)	P	OR (95% CI)	P	OR (95% CI)	P
Men (vs. Women)	0.42 (0.27-0.65)	<0.01	0.35 (0.15-0.81)	0.01	0.32 (0.14-0.72)	<0.01	0.30 (0.15-0.61)	<0.01
Age (per 1 year)	1.09 (1.07-1.12)	<0.01	1.10 (1.05-1.16)	<0.01	NA		NA	
Duration of diabetes (per 1 year)	1.05 (1.03-1.07)	<0.01	1.01 (0.98-1.16)	0.57	1.03 (1.00-1.06)	0.09	1.02 (1.00-1.05)	0.09
Current drinker (vs. None)	0.55 (0.33-0.88)	0.01	1.71 (0.73-4.06)	0.21	1.08 (0.49-2.37)	0.85	NA	
Smoking history (vs. Never)	0.78 (0.51-1.20)	0.26	NA		NA		NA	
Body mass index (per 1 kg/m^2^)	0.58 (0.51-0.64)	<0.01	0.52 (0.43-0.61)	<0.01	0.55 (0.47-0.63)	<0.01	0.55 (0.47-0.62)	<0.01
Antidiabetic medication								
Sulfonylureas	2.69 (1.36-5.22)	<0.01	1.11 (0.38-3.14)	0.85	NA		NA	
Metformin	1.06 (0.69-1.63)	0.78	NA		NA		NA	
Thiazolidinediones	3.28 (1.43-7.46)	<0.01	1.75 (0.54-5.72)	0.35	NA		NA	
DPP-4 inhibitors	3.00 (1.94-4.66)	<0.01	1.54 (0.76-3.14)	0.23	NA		NA	
SGLT2 inhibitors	0.51 (0.31-0.82)	<0.01	1.54 (0.69-3.51)	0.30	NA		NA	
GLP-1 receptor agonists	0.57 (0.28-1.06)	0.08	NA		NA		NA	
Insulin preparations	1.04 (0.64-1.66)	0.88	NA		NA		NA	
Hypertension	0.89 (0.55-1.47)	0.63	NA		NA		NA	
Hyper LDL-cholesterolemia	1.32 (0.84-2.09)	0.23	NA		NA		NA	
Hypo HDL-cholesterolemia	0.63 (0.33-1.11)	0.13	NA		NA		NA	
Diabetic retinopathy	1.36 (0.63-2.78)	0.42	NA		NA		NA	
Diabetic peripheral neuropathy	1.48 (0.94-2.35)	0.09	NA		NA		NA	
Albuminuria	0.88 (0.57-1.34)	0.55	NA		NA		NA	
Cerebrovascular disease	2.92 (1.60-5.26)	<0.01	7.14 (2.63-20.72)	<0.01	8.33 (3.23-22.65)	<0.01	7.18 (2.90-18.43)	<0.01
Coronary heart disease	1.16 (0.66-1.99)	0.59	NA		NA		NA	
Peripheral artery disease	1.33 (0.47-3.37)	0.57	NA		NA		NA	
Hemoglobin (per 10 g/L)	0.70 (0.61-0.80)	<0.01	1.15 (0.89-1.52)	0.28	1.08 (0.85-1.38)	0.54	1.08 (0.86-1.37)	0.51
Serum albumin (per 10 g/L)	0.41 (0.20-0.85)	0.02	0.98 (0.28-3.50)	0.98	0.53 (0.17-1.69)	0.29	0.54 (0.17-1.63)	0.27
LDL-cholesterol (per 1 mmol/L)	0.93 (0.73-1.18)	0.55	NA		NA		NA	
HDL-cholesterol (per 1 mmol/L)	3.05 (1.72-5.52)	<0.01	0.70 (0.28-1.79)	0.46	0.88 (0.35-2.18)	0.78	NA	
Uric acid (per 100 μmol/L)	0.47 (0.34-0.64)	<0.01	0.70 (0.43-1.13)	0.15	0.69 (0.43-1.10)	0.12	NA	
eGFR (per 10 mL/min/1.73 m^2^)	0.95 (0.86-1.04)	0.25	NA		NA		NA	
HbA1c (per 1%)	1.03 (0.93-1.13)	0.56	NA		NA		NA	
FIB-4 (per 1 unit)	1.98 (1.50-2.62)	<0.01	0.83 (0.48-1.42)	0.50	1.64 (1.06-2.58)	0.03	1.65 (1.07-2.56)	0.02

In the multiple logistic regression analysis (Model 1), which included variables that were significantly associated with low SMI in the univariate analysis, female sex, age, BMI, and history of cerebrovascular disease were identified as factors significantly associated with low SMI. However, contrary to the univariate analysis, the FIB-4 index was not significantly associated with low SMI in this model (OR 0.83, 95% CI 0.48-1.42, P=0.50). This discrepancy suggests that the effect of the FIB-4 index is masked when adjusted for age due to collinearity. The VIF for age and FIB-4 in the logistic regression model for low SMI was 1.67, indicating no significant multicollinearity. In Model 2, where age and age-related medication use (sulfonylureas, thiazolidinediones, DPP-4 inhibitors, and SGLT2 inhibitors) were excluded due to potential multicollinearity with FIB-4 (as age is a component of the FIB-4 formula), female sex, BMI, cerebrovascular disease, and the FIB-4 index remained significantly associated with low SMI. In Model 3, which included clinically relevant variables (sex, duration of diabetes, BMI, cerebrovascular disease, hemoglobin, serum albumin concentration, and the FIB-4 index), the FIB-4 index was also significantly associated with low SMI.

## Discussion

The principal finding of this study is that while the FIB-4 index was not significantly associated with SMI in the overall population after adjusting for age, it was significantly associated with low SMI specifically in non-elderly patients (<65 years). This suggests a potential link between liver fibrosis and altered muscle metabolism, which may be relevant to low muscle mass even at a pre-cirrhotic stage. In patients with type 2 diabetes, age and BMI are known determinants of SMI [4,6,7], and similar results have been obtained in our previous research [32].

Model 1 showed no significant association (OR 0.83, 95% CI 0.48-1.42, P=0.50), likely due to the inherent conceptual collinearity and the strong confounding effect of age (Figure 4). However, the FIB-4 index emerged as a significant factor when age was excluded (Model 2) or when variables were selected to minimize collinearity (Model 3). This suggests that while age-related confounding limits the FIB-4 index’s utility in the general population, it remains a relevant marker in younger subgroups where aging effects are less pronounced.

We also confirmed that the association between the FIB-4 index and low SMI remained significant even after adjusting for serum albumin (Models 2 and 3), suggesting the robustness of our findings. Incorporating the FIB-4 index, diabetes duration, and cerebrovascular history into clinical assessments may facilitate the early detection of muscle loss. Regarding pharmacotherapy, while SGLT2 inhibitors and GLP-1 receptor agonists have been reported to reduce the FIB-4 index [33-36], they may also affect muscle mass or increase fall risks [37-39]; thus, their use requires careful consideration based on patient characteristics.

It has been reported that MASLD is associated with sarcopenia [40] and dynapenia [41] in patients with type 2 diabetes. Our findings in the non-elderly group are consistent with reports by Sung et al. [21] and Kuchay et al. [22], which demonstrated a correlation between liver fibrosis markers and reduced skeletal muscle mass. Notably, the mean age in Kuchay’s cohort (53-55 years) aligns with our non-elderly subgroup. We extend these findings by clarifying the age-dependent nature of this relationship in a larger Japanese population. Our study clarifies the age-dependent relationship between SMI and the FIB-4 index. Although the interaction between age and the FIB-4 index was not statistically significant (χ^2^=1.44, P=0.13), likely due to limited power, exploratory subgroup analysis indicated a significant association specifically in non-elderly patients (<65 years). In the elderly, the influence of liver fibrosis may be masked by age-related physiological muscle loss, multiple comorbidities, and polypharmacy. Furthermore, the mathematical properties of the FIB-4 index, which inherently increase with age, may reduce its diagnostic specificity in older adults [42]. Thus, in patients under 65 years where aging effects are less pronounced, the FIB-4 index functions as an informative marker for early muscle mass loss.

In this study, a history of cerebrovascular disease was also significantly associated with low SMI. This can be easily understood as, in some cases with a history of cerebrovascular disease, a decrease in physical activity and impaired swallowing/eating function can lead to a reduction in skeletal muscle mass. On the other hand, the reason why diabetic peripheral neuropathy was not associated with low SMI is unknown. While diabetic neuropathy has been reported to be associated with a decrease in skeletal muscle mass [43,44], there are also reports that its impact is small in clinical practice due to the low number of severe cases [45]. The subjects of this study were outpatients with type 2 diabetes, and it is possible that there were few cases of malnutrition or reduced physical activity (judging from hemoglobin and serum albumin levels), which might have made the relationship between low SMI and neuropathy difficult to observe. Furthermore, the diagnosis of neuropathy was based on a simplified diagnostic criterion [31], and the lack of severity classification may also be a contributing factor.

This study has several limitations. First, this cross-sectional study precludes causal inference; low SMI may exacerbate systemic insulin resistance, thereby promoting liver fibrosis [46-48], but long-term prospective or intervention studies are needed to confirm the direction of this relationship. Furthermore, due to the retrospective nature of this study, we could not evaluate potential confounding factors such as physical activity, dietary intake, and inflammatory markers (e.g., C-reactive protein). Since these factors are known to influence both liver fibrosis and skeletal muscle mass [49,50], the inability to adjust for these unmeasured variables is a limitation of our study.

Second, the subjects were Japanese patients with type 2 diabetes at a single institution, so caution is needed when generalizing the results. Specifically, we excluded patients with severe comorbidities (e.g., advanced renal failure, malnutrition) to minimize confounding effects. However, these exclusion criteria may have introduced selection bias. For instance, excluding patients with thrombocytopenia (a key component of the FIB-4 formula) may have removed individuals with advanced fibrosis, while excluding those with malnutrition likely removed those with the lowest muscle mass. This "healthy user" effect likely attenuated the observed association between the FIB-4 index and SMI, suggesting that our results may be conservative estimates. Therefore, our findings should be interpreted with caution when applied to populations with advanced complications or severe frailty. Regarding liver disease etiologies, alcohol consumption status was assessed and is presented in Table 1. However, viral hepatitis screening or liver imaging was not systematically performed in all participants. Therefore, the potential influence of undiagnosed viral liver disease or other specific liver pathologies remains an unmeasured confounder.

Third, while the diagnosis of sarcopenia includes not only skeletal muscle mass but also the evaluation of muscle strength (e.g., handgrip strength) and physical performance (e.g., walking speed) [23], this study used only SMI as the main indicator of skeletal muscle mass. In the future, it is desirable to integrate data on muscle strength and physical performance to examine a more comprehensive association between sarcopenia and the FIB-4 index.

Fourth, while the FIB-4 index is an excellent non-invasive marker for liver fibrosis, its diagnostic accuracy does not match that of a liver biopsy.

Fifth, we did not account for the duration of antidiabetic medication use, particularly SGLT2 inhibitors and GLP-1 receptor agonists. The effects of these drugs on skeletal muscle mass may differ between newly initiated cases (early phase of treatment) and long-term users [51,52]. However, due to the cross-sectional nature of this study, we were unable to differentiate between these phases. Furthermore, a specific limitation regarding the FIB-4 index is its mathematical dependence on age. Since age is a component of the formula, the FIB-4 index values inherently increase with aging. This characteristic may complicate the interpretation of the contribution of liver fibrosis to muscle mass loss, particularly in older adults. Although we attempted to mitigate this by stratifying the analyses by age, we cannot entirely separate the effect of liver fibrosis from the profound confounding effect of age itself.

Finally, the interaction term between age and the FIB-4 index did not reach statistical significance (χ^2^=1.44, P=0.13). Therefore, the age-stratified results should be interpreted as exploratory. The lack of statistical significance for the interaction may be attributable to insufficient statistical power due to the limited sample size of the younger subgroup. Therefore, the age-stratified results must be strictly interpreted as exploratory and hypothesis-generating. Given these limitations, the independent clinical utility of the FIB-4 index for predicting muscle mass loss, distinct from the aging process, remains to be cautiously evaluated in future adequately powered studies.

To overcome these limitations and clarify the causal relationship between liver fibrosis and sarcopenia, it is necessary to conduct long-term prospective cohort and intervention studies. Specifically, studies that target patients with high the FIB-4 index and type 2 diabetes and track changes in SMI, muscle strength, physical function, and the FIB-4 index values after specific exercise or nutritional therapy interventions are desirable.

## Conclusions

Our study demonstrated an initial association between the FIB-4 index and low SMI in Japanese patients with type 2 diabetes. However, our multivariable analyses revealed that this association is largely driven by the profound confounding effect of age, which is an inherent mathematical component of the FIB-4 index scoring formula. Although our exploratory subgroup analyses suggest a potential signal specifically in non-elderly patients (aged <65 years), the lack of a statistically significant age interaction limits the ability to draw definitive conclusions. Therefore, the independent clinical utility of the FIB-4 index for assessing early muscle mass loss, distinct from the natural aging process, remains uncertain. Further adequately powered, long-term prospective cohort studies are required to clarify its role and validate its potential utility in specific clinical subpopulations.
